# Assessment of the genetic diversity of Atlantic bottlenose dolphin (*Tursiops truncatus*) strandings in the Mississippi Sound (USA)

**DOI:** 10.1371/journal.pone.0314249

**Published:** 2025-06-25

**Authors:** Mark A. Arick II, Nelmarie Landrau-Giovannetti, Chuan-Yu Hsu, Corrinne E. Grover, Stephen Reichley, Zenaida V. Magbanua, Olga Pechanova, Debra Moore, Ehsan Kayal, Anna Linhoss, Theresa Madrigal, Mark Peterman, Ozan Ozdemir, Daniel G. Peterson, Moby Solangi, Beth Peterman, Mark Lawrence, Attila Karsi

**Affiliations:** 1 Institute for Genomics, Biocomputing & Biotechnology, Mississippi State University, Mississippi State, Mississippi, United States of America; 2 Global Center for Aquatic Health and Food Security, Mississippi State University, Mississippi State, Mississippi, United States of America; 3 Department of Pathobiology and Population Medicine, College of Veterinary Medicine, Mississippi State University, Mississippi State, Mississippi, United States of America; 4 Department of Ecology, Evolution, and Organismal Biology, Iowa State University, Ames, Iowa, United States of America; 5 Department of Biosystems Engineering, College of Engineering, Auburn University, Auburn, Alabama, United States of America; 6 Institute for Marine Mammal Studies, Gulfport, Mississippi, United States of America; 7 Department of Comparative Biomedical Sciences, College of Veterinary Medicine, Mississippi State University, Mississippi State, Mississippi, United States of America; UNAM: Universidad Nacional Autonoma de Mexico, MEXICO

## Abstract

The common bottlenose dolphin (*Tursiops truncatus*) is a key marine mammal species in the Gulf of Mexico, playing an essential role as a top predator. This study investigates the genetic diversity and population structure of bottlenose dolphins stranded in the Mississippi Sound from 2010 to 2021. Tissue samples (muscle, liver, lung, kidney, and brain) were collected from 511 stranded dolphins, and mitochondrial DNAs (mtDNA) were extracted for analysis. A total of 417 samples were successfully amplified and sequenced using high throughput sequencing, yielding 386 complete mitogenomes. Genetic diversity metrics, such as nucleotide and haplotype diversity, were calculated, and population structure was inferred for both mitochondrial control region (mtCR) and whole mitogenome sequences. Using the whole mitogenome, the study identified four genetically distinct populations within the Mississippi Sound, demonstrating regional variation in dolphin populations. Notably, two stranded individuals likely originated from populations outside the sampled area. The use of whole mitogenomes allowed for improved resolution of genetic diversity and population differentiation compared to previous studies using partial mtDNA sequences. These findings enhance our understanding of bottlenose dolphin population structure in the region and underscore the value of stranded animals for population genetic studies.

## Introduction

*Tursiops truncatus* (common bottlenose dolphin) is the most abundant marine mammal in the Gulf of Mexico (GoMx), occupying diverse habitats, including estuarine, coastal, continental shelf and slope, and oceanic waters [1]. They are essential top-level predators and keystone species because of their impact on marine ecosystems. While common bottlenose dolphins are not considered endangered, they are a protected species in the United States under the Marine Mammal Protection Act (MMPA; 16 U.S.C 1361–1407) [2]. Under the provisions of the MMPA, the U.S. National Marine Fisheries Service (NMFS) must complete regular stock assessments of the species under its jurisdiction. They have designated 32 distinct bay, sound, and estuary (BSE) common bottlenose dolphin stocks in the northern GoMx, including the Mississippi Sound, Lake Borgne, and Bay Boudreau Common Bottlenose Dolphin stock. Based on a winter 2018 aerial survey, the best-known abundance estimate for common bottlenose dolphins in this stock is 1,265 individuals [2,3].

**Fig 1:**
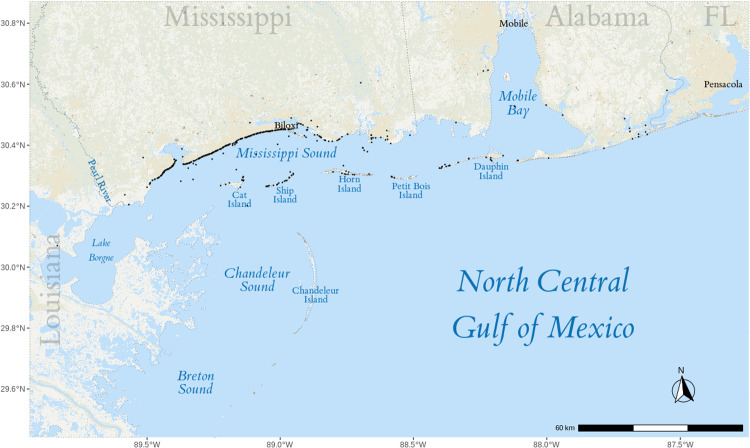
Map of sampled area. Map of the North Central Gulf of Mexico and the adjacent contiguous bodies of water, including the stranding locations of the sampled dolphins (black dots). The map was created using the tidyverse (v1.2.1) [14], sf (v1.0-18) [15,16], ggspatial (v1.1.9) [17], and stars (v0.6-6) [16] R libraries with data from the USGS 100-Meter Resolution Land Cover of the Conterminous United States.

Photo-identification, satellite telemetry, and genetic studies indicate that common bottlenose dolphins documented in Mississippi waters have been known to exhibit site fidelity, with some groups residing around the barrier islands of Mississippi (Cat Island, Ship Island, Horn Island, and Petit Bois Island) and others seasonally shifting from the inshore waters of the Mississippi Sounds to the adjacent contiguous bodies of water of the north central GoMx, Chandeleur Sound, Breton Sound, and Mobile Bay (Fig 1) [4–12]. Spatial analyses show that dolphin density is highest in the central and eastern portions of the Mississippi Sound [6]. Given these observed movement patterns, further genetic studies, particularly mitochondrial DNA (mtDNA) analysis from stranded individuals, are essential for distinguishing resident and transient populations.

Vollmer and Rosel’s study [13] on the population structure of *T. truncatus* in offshore and coastal waters of the US Gulf of Mexico identified seven genetically distinct populations, which contrasted with the five previous management stocks. The study highlighted that the geographic distribution of these populations correlates with various physiographic parameters, such as water depth and slope, indicating that environmental factors play a crucial role in shaping the population structure of bottlenose dolphins [13]. Three out of the seven identified stocks were classified as oceanic populations, which suggests that coastal populations of bottlenose dolphins exhibit more restricted dispersal compared to their oceanic counterparts. Coastal dolphins are often more affected by anthropogenic impacts, such as habitat degradation and pollution, which can lead to more significant genetic differentiation due to limited gene flow between populations. In contrast, oceanic dolphins may have broader ranges and more opportunities for gene flow, potentially resulting in less genetic differentiation among populations.

More recently, Vollmer et al. [11] analyzed the mitochondrial control region (mtCR) sequences and nuclear microsatellites from bottlenose dolphin samples in the Mississippi Sound and surrounding coastal waters. The study identified only two genetically distinct populations: one confined to the Mississippi Sound and adjacent coastal waters (green population) and another extending from Mobile Bay (Alabama) to Florida’s east coast (blue population) [11]. These findings emphasize the need for continued genetic assessments to refine stock delineations and inform conservation management.

Genetic diversity is crucial for the long-term survival of any given population. While cetaceans are highly mobile animals with significant dispersal potential, studies have identified various barriers to gene flow, leading to population sub-structuring in some species [18–20]. Understanding common bottlenose dolphins’ genetic diversity and population structure is essential for conservation, monitoring, and marine management projects. Consequently, there is an ever-increasing interest in applying molecular techniques such as single nucleotide polymorphisms (SNPs), mitochondrial DNA (mtDNA), nuclear microsatellites, and more recently, environmental DNA (eDNA), for identifying and monitoring populations of dolphins [21–26] for conservation purposes.

The analysis of stranded marine mammal carcasses provides an alternative to live-capture sampling, which can be costly and limited by sample size, sampling frequency, and seasonal constraints. Utilizing carcasses offers a valuable year-round tool for monitoring wild populations. However, several challenges exist. One major limitation is determining the source population of stranded animals, as ocean currents and wind can cause drift, complicating inferences about free-ranging populations. Some studies have attempted to model these effects to estimate origins [27–29]. Genetic sampling from carcasses is typically opportunistic, lacking a predetermined sample size or research area. The complexity of population structures further complicates efforts to identify the origins of stranded individuals [30].

Despite these challenges, stranded cetaceans have been successfully used for molecular species identification [31,32], assessments of genetic diversity [33,34], and population genetic structure [ 35,36], as well as for estimating demographic parameters [37]. In the Mississippi Sound, approximately 50 dolphin strandings are reported annually [2], with most classified as moderately autolyzed carcasses (code 3; [38]). Unlike the inconsistent availability of live tissues or fresh carcasses, this steady sample supply facilitates long-term genetic diversity and survivability surveys, particularly during environmental stressors such as natural and anthropogenic disturbances, climate variability, and disease outbreaks. Mitochondrial DNA (mtDNA) is especially useful for such studies due to its high abundance and relative resistance to degradation, allowing for the effective use of autolyzed tissues [39].

This study aims to enhance our understanding of the genetic structure of common bottlenose dolphins in the Mississippi Sound. Using whole mitochondrial genome sequences (mitogenomes), we investigate genetic variability among dolphins stranded along the Mississippi coast between 2010 and 2021. Despite the challenges of using stranded animals, this approach enables consistent annual sampling without the logistical constraints, financial burden, and stress imposed by live-capture methods. By leveraging the higher resolution of whole mitogenomes, we improve our ability to assess population structure within the Mississippi Sound.

## Materials and methods

### Sample collection

Common bottlenose dolphin tissues (327 muscle, 159 liver, 14 lung, 6 kidney, and 5 brain) were collected from 511 dolphins stranded in the Mississippi Sound from 2010 to 2021 by the Institute for Marine Mammals Studies (IMMS). The samples used in this study were from archived frozen (−20°C) tissues collected during necropsies. After sampling, the tissues were preserved by dropping small tissue pieces into 95% ethanol, which were then transferred to the Institute for Genomics, Biocomputing, & Biotechnology (IGBB) at Mississippi State University for analysis.

### Primer design

All complete mitogenomes for marine dolphins (family Delphinidae) available from NCBI RefSeq (S1 Table) were aligned against the complete common bottlenose dolphin (*T. truncatus*) mitogenome (NC_012059.1) using bwa fastmap (v0.7.10) [40] to find the set of longest pairwise exact matches. The coverage of all these sets of matches was calculated using bedtools (v2.25.0) [41]. Regions of the mitogenome with exact matches longer than 23 base pairs (bp) across the aligned genomes were considered candidate primer locations. Aided by Primer BLAST [42], these locations were manually screened, modified, and paired to produce candidate primer pairs (S2 Table).

### Extraction, amplification, and sequencing

Genomic DNA (gDNA) was extracted from the sampled tissues using the Qiagen DNeasy Blood & Tissue Kit (Qiagen, Germantown, MD, USA), following the manufacturer’s instructions. The quantity and purity of gDNA were assessed with a Nanodrop One spectrophotometer (Thermo Fisher Scientific, Waltham, MA, USA).

The complete mitogenome was amplified from extracted dolphin genomic DNA (20–30 ng) using four specific primer pairs (S2 Table) with overlapping amplicon regions and Phusion Hot Start Flex Master Mix (New England Biolabs, NEB, Ipswich, MA, USA). After AMpure XP beads (Beckman Coulter, Indianapolis IN, USA) cleanup, the amplicon pools (four amplicons per pool per sample) were used for Nanopore barcoded amplicon library preparation with Nanopore Ligation Sequencing Kit (SQK LSK109; Oxford Nanopore Technologies, Oxford, UK) and Native Barcoding Expansion 96 Kit (EXP-NBD196; Oxford Nanopore Technologies, Oxford, UK) and sequenced on a GridION sequencer (Oxford Nanopore Technologies, Oxford, UK) using a Flongle flow cell (FLO-FLG001; Oxford Nanopore Technologies, Oxford, UK).

After each sequencing run, the raw data were aligned to the common bottlenose dolphin reference mitogenome using minimap2 (v2.17-r941) [43,44]. The base depth for primary alignments was calculated using samtools (v1.9.4) [45], and the median base depth for each sample was graphed using R (v4.0.2) [46] with the tidyverse (v1.3.1) [14] package. Amplicons for samples with complete mitochondrial amplification but low coverage (median base depth less than or equal to 15) were resequenced.

Once the sequencing was complete, variant calling was performed with clair3 (v0.1-r10) [47] using the amplicon reference sequences to identify likely heteroplasmic samples. Samples with more than two high-quality (QUAL ≥ 20, i.e., 99% probability that a variant exists at the site; and GQ ≥ 20, i.e., 1% probability the call is incorrect) heterozygous SNPs were discarded. Medaka (v1.5.0) (https://github.com/nanoporetech/medaka) was used to make a consensus sequence for each amplicon in each sample using amplicon sequences parsed from the common bottlenose dolphin reference mitogenome. The amplicon sequences for each sample were assembled using CAP3 [48]. The complete mitogenomes were trimmed of overlap using merge, part of the EMBOSS tool suite (v6.6.0) [49], and rotated to start at the same base as the reference common bottlenose dolphin mitogenome using circlator (v1.5.5) [50]. To validate the likely species of each sample, the consensus sequence for each sample was aligned using BLAST+ (v2.14.0) [51] to the mitogenomes used during primer design (S1 Table). Any sample whose best alignment was a species other than *T. truncatus* was removed from further analysis.

Previously, genetic populations of bottlenose dolphins inhabiting the Mississippi Sound and coastal waters of the north-central Gulf of Mexico [11] and, more broadly, the coastal and offshore waters of the northern Gulf of Mexico [13] were studied using a portion of the mitochondrial control region (mtCR), along with other methods. To compare our samples to these two studies, the medaka polished amplicons that contained the mtCR region (P6) were reduced to the region between the primer pairs used to generate the published data (i.e., L15824 and H16265 or H16498; [52,53]) using cutadapt (v4.6) [54], discarding any sample without a match to L15824.

### mtCR and whole mitogenome analyses

The newly sequenced and previously published mtCR sequences (S3 Table) along with two *Stenella frontalis* mtCR sequences (DQ060054 and DQ060057) were aligned using mafft (v7.471) [55]. The mafft alignments were trimmed to remove gaps at either end of the alignments and any ambiguous base was resolved using the distribution of bases across all alignments using the R package ape (v5.7-1) [56]. The R package haplotypes (v1.1.3.1) [57] was used to condense the sequences into haplotypes. A Neighbor-joining tree was created using the R package ape (v5.7-1) [56] and visualized using tidyverse (v2.0.0) [14], ggtree (v3.6.2) [58], and aplot (v0.2.1) [59].

Both the principal component analysis (PCA) and population structure prediction were run using LEA (v3.0.0) [60–63] for K = 1 to K = 15 using the haplotypes after removing the outgroups. The population structure was run with ten repetitions per K. The percentage of variance explained for the principal components was calculated using the Tracy-Widom test [62,64] and used to generate a scree plot. The scree plot, using Cattell’s rule [65], and the lowest average cross-entropy were used to choose the best K (number of ancestral populations) for further analysis. The run (i.e., repetition) with the lowest cross-entropy from the selected K was graphed using tidyverse and used to group samples based on the highest admixture coefficient (q).

The nucleotide diversity (π), haplotype diversity, and population differentiation (Fst) were calculated for the trimmed dataset with the assigned group using the R packages pegas (v1.2) [66] and hierfstat (v0.5-11) [67]. An additional measure of population differentiation, ΦST, along with private alleles, was calculated using the R (v4.2.2) package poppr (v2.9.4) [68,69] with the adegenet (v2.1.10) [70,71] and ape (v5.7-1) [56] packages.

Similar methods were used to analyze the whole mitogenome sequences, albeit without the inclusion of published data and without trimming the mafft alignments. The complete mitogenome of *S. frontalis* (NC_060612.1) was used as an outgroup for phylogenetic analysis. Additionally, because the number of individuals assigned to each group was vastly different, the large groups were randomly sub-sampled into partitions roughly equal to the smallest group to calculate pairwise Fst and ΦST. For the partitioned groups, the average statistic across all partitions is reported.

## Results

### Data generation and quality control

Universal primer pairs were designed to amplify the mitogenome of marine dolphins (family Delphinidae) from 33 conserved regions ranging from 24 bp to 105 bp each. Ten candidate primer pairs were designed (P1 - P10; S2 Table; S1 Fig) and tested, resulting in the selection of four primer pairs (P4, P6, P7, and P10) as the best combination to retrieve the entire mitogenome of 16.3 Kbp.

Although most gDNAs were degraded, the extractions provided suitable templates for mtDNA amplification. Of the 511 samples collected, 417 had at least part of the mitogenome amplified (384 complete; 33 partial amplifications; S4 Table) from fresh dead (decomposition code 2; 66 individuals) to advanced decomposition (code 4; 33 individuals) samples, with most coming from moderately decomposed animals (code 3; 318 individuals). Notably, redundancy between primers P6, P7, and P10 (S1 Fig) resulted in fully amplified mitogenomes in six of the 33 samples with partial amplification. We identified 44 samples that did not pass the sequencing depth cutoff and required resequencing, with nine requiring multiple rounds to ensure data accuracy. The average median read coverage for the complete samples were 236.1, 322.1, 546.3, and 320.5 reads per sample for P4, P6, P7, and P10 primer pairs, respectively (S2 Fig).

Thirty-eight samples had at least one heterozygous variant; however, four (SER10–0256, SER11–0942, SER11–1021, and SER13–0420) were removed from further analysis due to excessive heterozygosity (4–37 heterozygous SNPs).Of the 413 remaining samples, all complete samples, along with six full-mtDNA partial samples, were assembled into whole, circular mitogenomes (16,387–16,405 bp). Smaller assemblies were generated for 27 samples (6,104–14,627 bp) where the entire mitogenome was not amplified. Of those, SER12–0678 was the only sample to recover only disjointed amplicons (i.e., P4 and P6).Since P6 encompasses most of the partial mtCR region used in previous studies [11,13] (ending approximately ten bases before the primer H16232), the ten samples lacking P6 (SER11–0040, SER11–1421, SER11–2252, SER11–2358, SER11–2425, SER12–0271, SER12–0735, SER13–0635, SER16–00070, and SER19–00351) were removed from further analysis. The P6 amplicon sequence in the remaining 403 samples was trimmed to the forward primer used in previous studies (L15824), producing an approximate 421 bp sequence for each sample.

### mtCR haplotypes

To compare our samples with those previously published, we first reconstructed mtCR haplotype using all samples combined. The 511 mtCR sequences (106 previously published, 403 newly sequenced, and 2 outgroups) were aligned using mafft (S3 Fig) and trimmed to 354 bases, representing 74 (72 bottlenose dolphin, 2 outgroups) haplotypes (sizes ranging from 1–198 members; S3 and S4 Tables), using the haplotypes R package. Of the 72 bottlenose dolphin haplotypes (S5 Table), 52 contain only published sequences (mtCR.pub), 12 contain both published and new sequences (mtCR.mix), and 8 contain only new sequences (mtCR.new). Almost half (190) of the new samples are contained within a single haplotype (mtCR.mix-1) and most (363) are contained within the top five haplotypes. Most of the mtCR.new groups are composed of single individuals (six of eight), with the remaining two (mtCR.new-1 and mtCR.new-2) containing three and two samples, respectively. Among the mtCR.new groups, all differ from an mtCR.mix group by a single nucleotide (S4 Fig).

**Fig 2:**
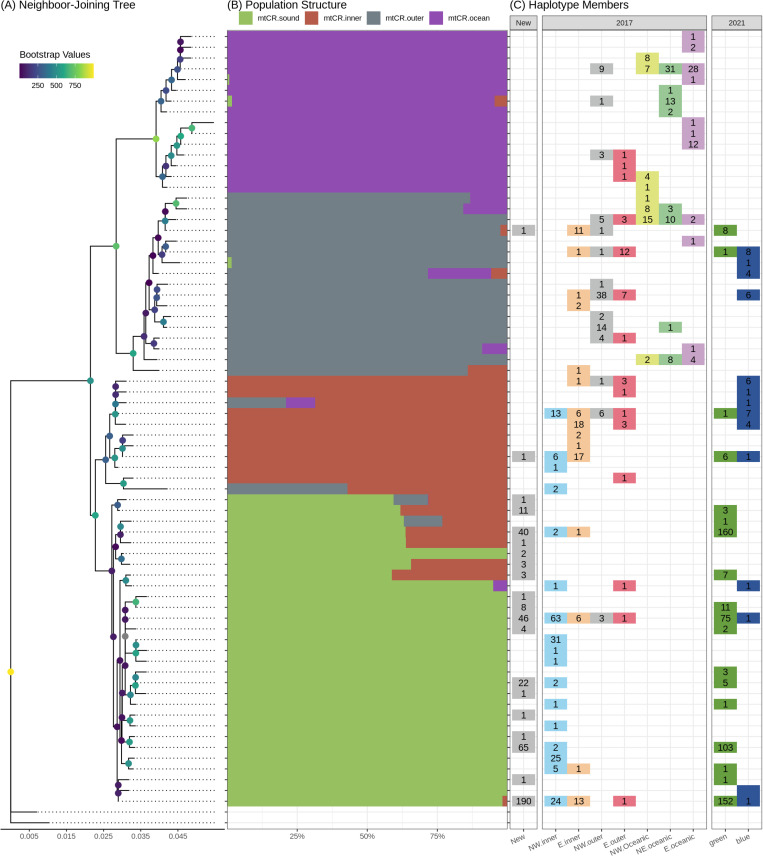
mtCR analyses. (A) Neighbor-joining tree, (B) LEA structural analysis (K = 4), and (C) the number of samples in each haplotype assigned to the populations described in Vollmer and Rosel [13] and Vollmer et al. [11].

A neighbor-joining tree was created using the 74 haplotypes (Fig 2A). After removing the outgroup sequences, LEA was used for the principal component and population structure analyses, evaluating up to 15 ancestral populations (K = 1 to K = 15) using the 44 biallelic SNPs present in the 72 haplotypes. Both the PCA scree plot (S5A Fig) and the cross-entropy plot (S5B Fig) suggest that four ancestral populations (K = 4) best describe the data (Fig 2B). Each haplotype was assigned a group based on the highest admixture coefficient (q). Additionally, the published samples assigned to populations described in Vollmer and Rosel [13] and Vollmer et al. [11] were linked to the current haplotypes (Fig 2C). As shown in S6 Table, the largest population (mtCR.sound) contains almost all (99.5%) newly sequence samples.

Overall haplotype diversity was 0.8083 (±2.012x10^-4^) among these samples, with haplotype diversity for each group ranging from 0.7562 (±2.671x10^-4^) in mtCR.sound to 0.9779 (±7.426x10^-4^) in mtCR.ocean. Overall nucleotide diversity (π) was 0.0113 (±3.939x10^-5^), and the per group nucleotide diversity ranges from 0.0037 (±6.681x10^-6^) for mtCR.sound and 0.0087 (±2.818x10^-5^) for mtCR.ocean. The overall differentiation (Fst) was 0.0627 using the Weir and Cockerham method [72], with pairwise Fst (S7 Table) ranging from 0.0296 (mtCR.inner vs mtCR.sound) to 0.0867 (mtCR.sound vs mtCR.ocean). The overall ΦST was 0.6181. The pairwise ΦST (S7 Table) ranged from 0.4239 (mtCR.inner vs mtCR.outer) to 0.7442 (mtCR.ocean vs mtCR.sound). All four groups have private alleles, ranging from five for mtCR.inner to nine in mtCR.sound.

### Full mitogenome

Because the full mitogenome may offer additional resolution [73–75], the 386 complete mitogenomes and the outgroup mitogenome from *S. frontalis* were aligned using mafft, resulting in an alignment length of 16,508 bases. These 386 common bottlenose dolphin sequences were condensed into 117 haplotypes, most (84) of which comprise single individuals. As expected, these haplotypes are consistent with the mtCR haplotypes but with additional resolution (S6 Fig).

**Fig 3:**
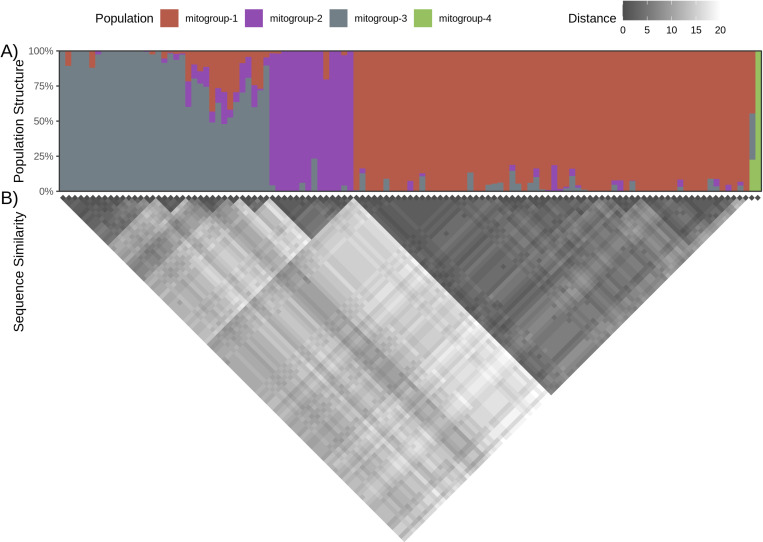
Mitogenome population structure. A) LEA population structure analysis (K = 4). Each bar represents one of the whole mitogenome haplotypes with each segment showing the admixture coefficient (q) of the four inferred populations in the haplotype. B) Sequence similarity of the whole mitogenome haplotypes. Each cell shows the number of differences between two haplotypes. The dark triangles are groups of haplotypes with high sequence similarity.

PCA and putative population structure were analyzed (via LEA) using 309 biallelic SNPs identified among the 117 haplotypes. The PCA scree plot (S7A Fig) and the minimum cross-entropy plot (S7B Fig) suggest that the data represent four populations (K = 4, hereafter mitogroups 1–4; Fig 3A). As with the mtCR region, each haplotype was assigned to a mitogroup based on the maximum admixture coefficient (q), clustering 237 samples (67 haplotypes) in mitogroup 1; 35 samples (14 haplotypes) in mitogroup 2; 113 samples (35 haplotypes) in mitogroup 3; and 1 sample (1 haplotype) in mitogroup 4. The neighbor-joining tree (S8 Fig) is largely congruent with the structure analysis. Given the admixture coefficients (S8 Table) of the included samples show two distinct subgroups in mitogroup 3, it is not unsurprising that this group is paraphyletic; which could suggest a large ancestral population that has separated over time.

Only two haplotypes (mito-097, q = 0.9997, and mito-062, q = 0.2250) had an admixture coefficient in mitogroup 4, with mito-097 being the only haplotype assigned to that mitogroup. Notably, the two sequences represented by these two haplotypes are the only two samples not assigned to the mtCR.sound group in the mtCR analysis; i.e., SER11–0141 was assigned to mtCR.inner and SER19–00888 to mtCR.outer. Additionally, these two haplotypes are unusually dissimilar compared to the other sequences (Fig 3B) and, in the neighbor-joining tree, these two haplotypes are separated from the other clades (S8 Fig). Taken together, these two samples likely originated from population outside the sampled area. Since mitogroup 4 only contained a single haplotype, it was dropped from most downstream analyses.

**Fig 4:**
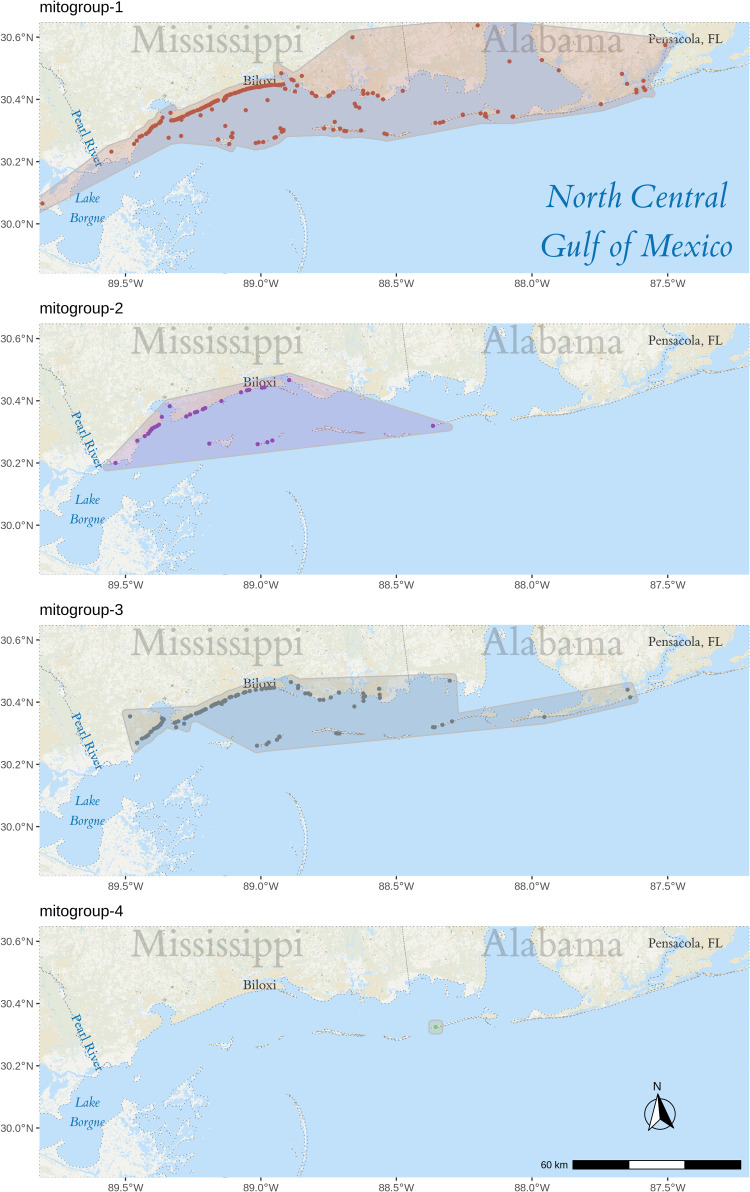
Stranding locations per mitogroup. Map of the stranding location for each group. Map created using the tidyverse (v1.2.1) [14], sf (v1.0-18) [15,16], ggspatial (v1.1.9) [17], and stars (v0.6-6) [16] R libraries with data from the USGS 100-Meter Resolution Land Cover of the Conterminous United States.

The stranding locations per group are shown in (Fig 4). The stranding location areas of group 2 and 3 are contained within that of group 1, with group 1 extending as far east as Pensacola, FL and as far west as Lake Borgne. Interestingly, the stranding region of group 2 is noticeably smaller than that of both group 1 and 3, extending from the Pearl River to Biloxi, MS. To test if the smaller region is due to the smaller population size, a bootstrap test with 1,000,000 iterations was run against the mean longitude using the boot (v1.3-30) [76,77] and boot.pval (v0.5) [78] R libraries. P-values were calculated using the normal approximation confidence intervals. The mean longitude of groups 1 and 2 are significantly different (p-values: 0.00437 and < 10^−6^, respectively) from the entire dataset.

The overall haplotype diversity was 0.9363 (±6.34x10^-5^), while the individual group haplotype diversity was 0.8604 (±3.70x10^-4^), 0.8857 (±8.46x10^-4^), and 0.8761 (±4.24x10^-4^) for mitogroups 1–3, respectively. The nucleotide diversity (π) was 0.00051 (±6.77x10^-8^) overall and 0.00017 (±1.01x10^-8^), 0.00018 (±1.15x10^-8^), and 0.00031 (±2.80x10^-8^) for mitogroups 1, 2, and 3, respectively. Since both mitogroups 1 and 3 are much larger than group 2, the pairwise population differentiation statistics Fst and ΦST were calculated on 6 and 3 subsets, respectively, and averaged for each comparison that included either of those two groups. Overall Fst and ΦST were 0.5784 and 0.7250, respectively. Pairwise differentiation ranges from 0.6475 (between mitogroups 1 and 3) to 0.8612 (mitogroups 2 and 4) for Fst and 0.6646 (between 1 and 3) and 0.8612 (between 2 and 4) for ΦST. Only mitogroup 1 had any private alleles.

## Discussion

Using the assembly methods described, the overlapping region added by P10 is superfluous and can be omitted. We obtained complete mitogenomes from stranded samples ranging from fresh dead (code 2) to advanced decomposition (code 4), with most of the samples being moderately decomposed (code 3). Interestingly, the decomposition does not seem to affect the likelihood of successful mtDNA amplification. We excluded mummified (code 5) remains; however, with current method advancements [79] in hard tissue DNA extraction, those samples could be an additional source of mtDNA to consider.

It is worth noting that our study used a methodology different from that of Vollmer et al. [11] and Vollmer and Rosel [13]. These two previous studies used skin samples from live common bottlenose dolphins and a combination of nuclear genomic sources and part of the mitochondrial control-region (mtCR). Since the newly sequenced samples were collected from stranded dolphins at several stages of decomposition, the extracted DNA was often highly degraded and contained high levels of bacterial contamination. While it is possible to analyze small allele microsatellites (<200 bp) using highly degraded DNA [80], some of the microsatellites targeted in previous studies do not fit into this category. Since it was likely we would not be able to use some of the microsatellites used in previous studies due to the decomposition level of many samples, we focused on amplifying the entire mitogenome. By using the entire mitogenome, we increased the number of usable SNPs identified (309 vs 44), allowing for more precision in identifying populations (the single population containing almost all newly sequenced haplotypes in Fig 2B vs the three groups in Fig 3A), while simultaneously reducing the impact of homoplasy, which is common in the mtCR. Previous research in marine animals has demonstrated the value in using mitogenomes to analyze complex evolutionary relationships. For example, Leslie, Archer, and Morin [73] used whole mitogenome sequences from spinner (*Stenella longirostris*) and pantropical spotted dolphins (*Stenella atteunuata*) to test population structure hypotheses at multiple hierarchical taxonomic levels, although the ability to distinguish among populations units within each species was interestingly not improved from earlier studies by the inclusion of the entire mitogenome. Conversely, other investigators [74] used next-generation sequencing (NGS) to generate a total of over 500 whole mitogenomes from seven cetacean species and green sea turtles, showing that entire mitogenomes exhibited greater variation than shorter regions, thereby improving resolution. Likewise, Nykänen et al. [75] found entire mitogenomes improved resolution in reconstructing post-glacial expansion of the Northeast Atlantic bottlenose dolphins. While these and other studies clearly demonstrate the utility of mitogenomes in providing insight into marine populations, it is important to note that, although mtDNA sequencing can provide valuable insight into genetic diversity and migration, it only provides information about the maternal lineage and may not reflect the overall genetic diversity of a population, and our study would benefit from additional nuclear genomic sources. Given the decreasing cost and improving quality of long-read sequencing technologies, future studies will benefit from scaling up to whole mitogenome as well as including nuclear markers, such as SNPs or microsatellites. Additionally, given the large overlap between the four amplicons, it may be possible to generate and include the multiple genomes of heteroplasmic samples in the structural and comparative analyses.

Looking at the entire northern GoMx, Vollmer and Rosel [13] reported seven populations (Northwest Inner Shelf, Northwest Outer Shelf, Northwest Oceanic, East Inner Shelf, East Outer Shelf, East Oceanic, and Northeast Oceanic) based on the population structure analysis of the mtCR, microsatellites, and nuclear genomics SNPs. When using only the mtCR (along with adding the newly sequenced samples), four populations (mtCR.sound, mtCR.inner, mtCR.outer, and mtCR.oceanic) were found. The mtCR.sound population contained almost all haplotypes with new samples, as well as the majority (88%) of the previously described Northwest Inner Shelf [13] population, mtCR.inner contains a majority (55%) of the East Inner Shelf and some (12%) Northwest Inner Shelf sequences, and mtCR.outer contains a majority (61%) of the East Outer Shelf and Northwest outer sequences, as well as more than half (57%) of Northwest Oceanic sequences. The mtCR.ocean group largely consists of oceanic populations from Northeast, East, and Northwest. Additionally, Vollmer et al. [11] focused on the Mississippi Sound and surrounding coastal waters, reporting two populations (Green, encompassing the Mississippi Sound, and Blue, coastal waters east of Mobile Bay). Almost all (97%) of the Green population are assigned to mtCR.sound. The samples for the smaller Blue population are evenly split between the mtCR.inner (47%) and mtCR.outer (44%) populations and is largely absent from both mtCR.sound (9%) and mtCR.ocean (0%). The overall haplotype diversity falls within the ranges reported in Vollmer and Rosel [13] (0.6916–0.8633) and Vollmer et al. [11] (0.7816–0.8943); however, the per-group haplotype diversity range extends higher. Overall nucleotide diversity (pi) is on the lower end of the ranges reported in Vollmer and Rosel [13] (0.0062–0.0200) and Vollmer et al. [11] (0.0053–0.0202). The Fst values are lower than the values reported in Vollmer and Rosel [13] (0.18 overall Fst) and Vollmer et al. [11] (0.16 overall Fst); however, this is not unexpected since the populations inferred using LEA combined several of the published populations. Overall ΦST falls between the values reported in Vollmer and Rosel [13] (0.55) and Vollmer et al. [11] (0.65). Pairwise ΦST is within the range in Vollmer and Rosel [13] (0.041–0.799), but higher than reported in Vollmer et al. [11] (0.36–0.51). Taken together, the structure appears to transition from Green/NW inner (mtCR.sound), to Blue/Inner Shelf (mtCR.inner), then Blue/Outer Shelf/NW oceanic (mtCR.outer), and finally oceanic (mtCR.ocean) populations. The member haplotypes of each group are largely congruent with the populations described in both Vollmer and Rosel [13] and Vollmer et al. [11]; however, since the structure analysis did not include the microsatellite or SNP data, some of the published populations were combined. Additionally, the mtCR analysis suggests a single group (mtCR.sound) that commonly strands along the Mississippi coast, corresponding to the green population in Vollmer et al. [11] and the Northwest inner population in Vollmer and Rosel [13], with two samples likely originating outside the Mississippi Sound.

By comparison, whole mitogenome structure analysis and differentiation statistics also suggest two samples originated outside the sample area, but this method defines a slightly more complex system for the Mississippi Sound, with two groups that commonly strand across the entire sampled region (mitogroup 1 and 3) and a third group (mitogroup 2) that occasionally strands in a specific subset (between the Biloxi Bay and Pearl River). Some species of marine mammals show genetic differentiation over small geographic scales, despite the fact that they are generally considered to have a high propensity to disperse; e.g., Indo-Pacific bottlenose dolphins (*T. aduncus*) [81], Commerson’s dolphins (*Cephalorhynchus commersonii*) [82], and harbor porpoises (*Phocoena phocoena*) [83]. Unfortunately, the region each population inhabits is difficult to interpret due to the disjunction between the actual habitat utilized while alive and the post-mortem stranding location, the latter of which is influenced by factors such as sea currents and the unpredictable behavior of dying dolphins; however, the quantity of mitochondrial diversity recovered in the present study suggests there are three different subpopulations of common bottlenose dolphins in the Mississippi Sound area, providing finer-scale resolution of genetic structure among these mammals than previously reported.

Our research demonstrates that whole mitogenome sequences derived from various tissues sampled from stranded carcasses, with muscle and liver being best, may be suitable for characterizing and monitoring the genetic diversity of cetaceans in areas like the Mississippi Sound. It also provides important insights into the genetic diversity of common bottlenose dolphins in the Mississippi Sound, which can help inform conservation efforts and management decisions for this population.

## Supporting information

S1 Table Accession number and species of mitochondrial genomes used in primer design. (XLSX)

S2 Table Tested primer pairs, highlighted pairs were used in final analysis. (XLSX)

S3 Table Previously sequenced mtCR haplotypes of GoMx bottlenose dolphins with the new haplotype and assigned group, along with the population assignments in Vollmer and Rosel [13] and Vollmer et al [11]. (XLSX)

S4 Table Information of sequenced samples from this study. (XLSX)

S5 Table Summary of mtCR haplotypes. (XLSX)

S6 Table Comparison of membership sample and haplotype totals of each mtCR inferred population and the reported population in Vollmer and Rosel [13] and Vollmer et al. [11]. (XLSX)

S7 Table Population Statistics. (XLSX)

S8 Table Full mitogenome LEA admixture for the run with the lowest cross entropy using K = 4. (XLSX)

S1 Fig Tested primer pair locations on the mitochondrial genome. Highlighted pairs (P4, P6, P7, and P10) were used to amplify the mtDNA in all samples.
(TIF)

S2 Fig Median base coverage across each amplicon for each sample. (TIF)

S3 Fig Alignment of all mtCR sequences (both published and newly sequenced). Samples are shown in rows, alignment position is shown in columns. The missing data (white) at both ends were trimmed.
(TIF)

S4 Fig Nucleotide differences between the mtCR.new haplotypes and the most similar sequences. All sequences with a single mismatched base to the haplotype of interest are displayed. All identical bases are removed for clarity.
(TIF)

S5 Fig PCA Scree plot (A) and Minimum cross-entropy per K (B) for the mtCR structure analysis. (TIF)

S6 Fig Sankey plot showing the relationship between the haplotypes from mtCR to whole mitogenome. Haplotypes with five or more members are labeled.
(TIF)

S7 Fig PCA Scree plot (A) and Minimum cross-entropy per K (B) for the whole mitogenome structure analysis. (TIF)

S8 Fig Neighbor-joining tree for the whole mitogenome. Outgroup is the *S. frontalis* sequence NC_060612.1. Color shoe the assigned group of each sample.
(TIF)

## References

[1] Vollmer NL, Rosel PE. A Review of Common Bottlenose Dolphins (*Tursiops truncatus truncatus*) in the Northern Gulf of Mexico: Population Biology, Potential Threats, and Management. Southeast Nat. 2013;12:1–43. doi: 10.1656/058.012.m601

[2] Hayes SH. U.S. Atlantic and Gulf of Mexico Marine Mammal Stock Assessments 2021. 2022 [cited 13 Jun 2024]. doi: 10.25923/6TT7-KC16

[3] Garrison LP, Ortega-Ortiz J, Rappucci G. Abundance of Coastal and Continental Shelf Stocks of Common Bottlenose and Atlantic Spotted Dolphins in the Northern Gulf of Mexico: 2017-2018. 2021 [cited 13 Jun 2024]. doi: 10.25923/VK95-T881

[4] Hubard CW, Maze-Foley K, Mullin KD, Schroeder WW. Seasonal Abundance and Site Fidelity of Bottlenose Dolphins (*Tursiops truncatus*) in Mississippi Sound. Aquat Mamm. 2004;30(2):299–310. doi: 10.1578/am.30.2.2004.299

[5] Mullin KD, McDonald T, Wells RS, Balmer BC, Speakman T, Sinclair C, et al. Density, abundance, survival, and ranging patterns of common bottlenose dolphins (*Tursiops truncatus*) in Mississippi Sound following the Deepwater Horizon oil spill. PLoS One. 2017;12(10):e0186265. doi: 10.1371/journal.pone.0186265 29053728 PMC5650146

[6] Pitchford JL, Pulis EE, Evans K, Shelley JK, Serafin BJS, Solangi M. Seasonal density estimates of *Tursiops truncatus* (Bottlenose dolphin) in the Mississippi Sound from 2011 to 2013. Southeast Nat. 2016;15:188–206.

[7] Pitchford JL, Howard VA, Shelley JK, Serafin BJS, Coleman AT, Solangi M. Predictive spatial modelling of seasonal bottlenose dolphin (*Tursiops truncatus*) distributions in the Mississippi Sound. Aquat Conserv Mar Freshw Ecosyst. 2015;26(2):289–306. doi: 10.1002/aqc.2547

[8] Rosel P, Wilcox L, Sinclair C, Speakman T, Tumlin M, Litz J, et al. Genetic assignment to stock of stranded common bottlenose dolphins in southeastern Louisiana after the Deepwater Horizon oil spill. Endang Species Res. 2017;33:221–34. doi: 10.3354/esr00780

[9] Samuelson MM, Fujiwara M, Pulis EE, Pitchford J, Howard VA, Solangi M. Comprehensive Evaluation of Survival and Population Growth for Common Bottlenose Dolphins (*Tursiops Truncatus*) in the Mississippi Sound, Usa, Following the Deepwater Horizon Oil Spill. Southeast Nat. 2021;20(1). doi: 10.1656/058.020.0121

[10] Tyson R, Nowacek S, Nowacek D. Community structure and abundance of bottlenose dolphins *Tursiops truncatus* in coastal waters of the northeast Gulf of Mexico. Mar Ecol Prog Ser. 2011;438:253–65. doi: 10.3354/meps09292

[11] Vollmer NL, Rosel PE, Mullin KD, Schwacke LH, Garrison LP, Balmer BC, et al. Assessing common bottlenose dolphin (*Tursiops truncatus*) population structure in Mississippi Sound and coastal waters of the north central Gulf of Mexico. Aquatic Conservation. 2021;31(10):2951–66. doi: 10.1002/aqc.3668

[12] Wells R, Schwacke L, Rowles T, Balmer B, Zolman E, Speakman T, et al. Ranging patterns of common bottlenose dolphins *Tursiops truncatus* in Barataria Bay, Louisiana, following the Deepwater Horizon oil spill. Endang Species Res. 2017;33:159–80. doi: 10.3354/esr00732

[13] Vollmer NL, Rosel PE. Fine-scale population structure of common bottlenose dolphins (*Tursiops truncatus*) in offshore and coastal waters of the US Gulf of Mexico. Mar Biol. 2017;164(8). doi: 10.1007/s00227-017-3186-x

[14] Wickham H, Averick M, Bryan J, Chang W, McGowan L, François R, et al. Welcome to the Tidyverse. J Open Source Softw. 2019;4(43):1686. doi: 10.21105/joss.01686

[15] Pebesma E. Simple Features for R: Standardized Support for Spatial Vector Data. The R Journal. 2018;10(1):439. doi: 10.32614/rj-2018-009

[16] Pebesma E, Bivand R. Spatial data science: With applications in R. Chapman and Hall/CRC; 2023. doi: 10.1201/9780429459016

[17] Dunnington D. ggspatial: Spatial data framework for ggplot2. 2023. Available: https://CRAN.R-project.org/package=ggspatial

[18] Gaspari S, Holcer D, Mackelworth P, Fortuna C, Frantzis A, Genov T, et al. Population genetic structure of common bottlenose dolphins (*Tursiops truncatus*) in the Adriatic Sea and contiguous regions: implications for international conservation. Aquat Conserv Mar Freshw Ecosyst. 2013;25(2):212–22. doi: 10.1002/aqc.2415

[19] Louis M, Viricel A, Lucas T, Peltier H, Alfonsi E, Berrow S, et al. Habitat-driven population structure of bottlenose dolphins, *Tursiops truncatus*, in the North-East Atlantic. Mol Ecol. 2014;23(4):857–74. doi: 10.1111/mec.12653 24383934

[20] Quérouil S, Silva MA, Freitas L, Prieto R, Magalhães S, Dinis A, et al. High gene flow in oceanic bottlenose dolphins (*Tursiops truncatus*) of the North Atlantic. Conserv Genet. 2007;8(6):1405–19. doi: 10.1007/s10592-007-9291-5

[21] Adams CIM, Knapp M, Gemmell NJ, Jeunen G-J, Bunce M, Lamare MD, et al. Beyond Biodiversity: Can Environmental DNA (eDNA) Cut It as a Population Genetics Tool? Genes (Basel). 2019;10(3):192. doi: 10.3390/genes10030192 30832286 PMC6470983

[22] Hunter M, Meigs-Friend G, Ferrante J, Takoukam Kamla A, Dorazio R, Keith-Diagne L, et al. Surveys of environmental DNA (eDNA): a new approach to estimate occurrence in Vulnerable manatee populations. Endang Species Res. 2018;35:101–11. doi: 10.3354/esr00880

[23] Martien KK, Baird RW, Hedrick NM, Gorgone AM, Thieleking JL, McSweeney DJ, et al. Population structure of island‐associated dolphins: Evidence from mitochondrial and microsatellite markers for common bottlenose dolphins (*Tursiops truncatus*) around the main Hawaiian Islands. Mar Mammal Sci. 2011;28(3). doi: 10.1111/j.1748-7692.2011.00506.x

[24] Morin P, Archer F, Pease V, Hancock-Hanser B, Robertson K, Huebinger R, et al. Empirical comparison of single nucleotide polymorphisms and microsatellites for population and demographic analyses of bowhead whales. Endang Species Res. 2012;19(2):129–47. doi: 10.3354/esr00459

[25] Sellas AB, Wells RS, Rosel PE. Mitochondrial and nuclear DNA analyses reveal fine scale geographic structure in bottlenose dolphins (*Tursiops truncatus*) in the Gulf of Mexico. Conserv Genet. 2005;6(5):715–28. doi: 10.1007/s10592-005-9031-7

[26] Valsecchi E, Coppola E, Pires R, Parmegiani A, Casiraghi M, Galli P, et al. A species-specific qPCR assay provides novel insight into range expansion of the Mediterranean monk seal (*Monachus monachus*) by means of eDNA analysis. Biodivers Conserv. 2022;31(4):1175–96. doi: 10.1007/s10531-022-02382-0

[27] Peltier H, Dabin W, Daniel P, Van Canneyt O, Dorémus G, Huon M, et al. The significance of stranding data as indicators of cetacean populations at sea: Modelling the drift of cetacean carcasses. Ecol Indic. 2012;18:278–90. doi: 10.1016/j.ecolind.2011.11.014

[28] Nero R, Cook M, Coleman A, Solangi M, Hardy R. Using an ocean model to predict likely drift tracks of sea turtle carcasses in the north central Gulf of Mexico. Endang Species Res. 2013;21(3):191–203. doi: 10.3354/esr00516

[29] Shahidzadehasadi M, Linhoss A, Moore D, Reichley S, Mickle P, Lawrence M. Examining the effect of salinity on dolphin mortality using Lagrangian particle tracking in a hydrodynamic model. Estuar Coast Shelf Sci. 2024;297:108605. doi: 10.1016/j.ecss.2023.108605

[30] Hohn A, Thomas L, Carmichael R, Litz J, Clemons-Chevis C, Shippee S, et al. Assigning stranded bottlenose dolphins to source stocks using stable isotope ratios following the Deepwater Horizon oil spill. Endang Species Res. 2017;33:235–52. doi: 10.3354/esr00783

[31] Cypriano‐Souza AL, de Meirelles ACO, Carvalho VL, Bonatto SL. Rare or cryptic? The first report of an Omura’s whale (*Balaenoptera omurai*) in the South Atlantic Ocean. Marine Mammal Science. 2016;33(1):80–95. doi: 10.1111/mms.12348

[32] Siciliano S, Valiati VH, Emin-Lima R, Costa AF, Sartor J, Dorneles T, et al. New genetic data extend the range of river dolphins Inia in the Amazon Delta. Hydrobiologia. 2016;777(1):255–69. doi: 10.1007/s10750-016-2794-7

[33] Dalebout ML, Robertson KM, Frantzis A, Engelhaupt D, Mignucci-Giannoni AA, Rosario-Delestre RJ, et al. Worldwide structure of mtDNA diversity among Cuvier’s beaked whales (*Ziphius cavirostris*): implications for threatened populations. Mol Ecol. 2005;14(11):3353–71. doi: 10.1111/j.1365-294X.2005.02676.x 16156808

[34] Silva VS, Skueresky N, Lopes F, Koch TK, Ott PH, Siciliano S, et al. Integrating morphology and DNA barcoding to assess cetacean diversity in Brazil. Mamm Res. 2021;66(2):349–69. doi: 10.1007/s13364-021-00555-w

[35] Bilgmann K, Möller LM, Harcourt RG, Kemper CM, Beheregaray LB. The use of carcasses for the analysis of cetacean population genetic structure: a comparative study in two dolphin species. PLoS One. 2011;6(5):e20103. doi: 10.1371/journal.pone.0020103 21655285 PMC3105009

[36] Natoli A, Birkun A, Aguilar A, Lopez A, Hoelzel AR. Habitat structure and the dispersal of male and female bottlenose dolphins (*Tursiops truncatus*). Proc Biol Sci. 2005;272(1569):1217–26. doi: 10.1098/rspb.2005.3076 16024385 PMC1564106

[37] Schwacke L, Thomas L, Wells R, McFee W, Hohn A, Mullin K, et al. Quantifying injury to common bottlenose dolphins from the Deepwater Horizon oil spill using an age-, sex- and class-structured population model. Endang Species Res. 2017;33:265–79. doi: 10.3354/esr00777

[38] Geraci JR, Lounsbury VJ. Marine Mammals Ashore: A Field Guide for Strandings. National Aquarium in Baltimore; 2005.

[39] Foran DR. Relative degradation of nuclear and mitochondrial DNA: an experimental approach. J Forensic Sci. 2006;51(4):766–70. doi: 10.1111/j.1556-4029.2006.00176.x 16882217

[40] Li H. Aligning sequence reads, clone sequences and assembly contigs with BWA-MEM. 2013 [cited 9 Feb 2024]. doi: 10.48550/ARXIV.1303.3997

[41] Quinlan AR, Hall IM. BEDTools: a flexible suite of utilities for comparing genomic features. Bioinformatics. 2010;26(6):841–2. doi: 10.1093/bioinformatics/btq033 20110278 PMC2832824

[42] Ye J, Coulouris G, Zaretskaya I, Cutcutache I, Rozen S, Madden TL. Primer-BLAST: a tool to design target-specific primers for polymerase chain reaction. BMC Bioinformatics. 2012;13:134. doi: 10.1186/1471-2105-13-134 22708584 PMC3412702

[43] Li H. Minimap2: pairwise alignment for nucleotide sequences. Bioinformatics. 2018;34(18):3094–100. doi: 10.1093/bioinformatics/bty191 29750242 PMC6137996

[44] Li H. New strategies to improve minimap2 alignment accuracy. Bioinformatics. 2021;37(23):4572–4. doi: 10.1093/bioinformatics/btab705 34623391 PMC8652018

[45] Danecek P, Bonfield JK, Liddle J, Marshall J, Ohan V, Pollard MO, et al. Twelve years of SAMtools and BCFtools. Gigascience. 2021;10(2):giab008. doi: 10.1093/gigascience/giab008 33590861 PMC7931819

[46] R Core Team. R: a language and environment for statistical computing. Vienna, Austria: R Foundation for Statistical Computing; 2022. Available: https://www.R-project.org/

[47] Zheng Z, Li S, Su J, Leung AW-S, Lam T-W, Luo R. Symphonizing pileup and full-alignment for deep learning-based long-read variant calling. 2021. doi: 10.1101/2021.12.29.474431 38177392

[48] Huang X, Madan A. CAP3: A DNA sequence assembly program. Genome Res. 1999;9(9):868–77. doi: 10.1101/gr.9.9.868 10508846 PMC310812

[49] Rice P, Longden I, Bleasby A. EMBOSS: the European Molecular Biology Open Software Suite. Trends Genet. 2000;16(6):276–7. doi: 10.1016/s0168-9525(00)02024-2 10827456

[50] Hunt M, Silva ND, Otto TD, Parkhill J, Keane JA, Harris SR. Circlator: automated circularization of genome assemblies using long sequencing reads. Genome Biol. 2015;16:294. doi: 10.1186/s13059-015-0849-0 26714481 PMC4699355

[51] Camacho C, Coulouris G, Avagyan V, Ma N, Papadopoulos J, Bealer K, et al. BLAST+: architecture and applications. BMC Bioinformatics. 2009;10:421. doi: 10.1186/1471-2105-10-421 20003500 PMC2803857

[52] Rosel PE, Dizon AE, Heyning JE. Genetic analysis of sympatric morphotypes of common dolphins (genus *Delphinus*). Marine Biology. 1994;119(2):159–67. doi: 10.1007/bf00349552

[53] Rosel PE, Tiedemann R, Walton M. Genetic evidence for limited trans-Atlantic movements of the harbor porpoise *Phocoena phocoena*. Marine Biology. 1999;133(4):583–91. doi: 10.1007/s002270050498

[54] Martin M. Cutadapt removes adapter sequences from high-throughput sequencing reads. EMBnet J. 2011;17(1):10. doi: 10.14806/ej.17.1.200

[55] Katoh K, Standley DM. MAFFT multiple sequence alignment software version 7: improvements in performance and usability. Mol Biol Evol. 2013;30(4):772–80. doi: 10.1093/molbev/mst010 23329690 PMC3603318

[56] Paradis E, Schliep K. ape 5.0: an environment for modern phylogenetics and evolutionary analyses in R. Bioinformatics. 2019;35(3):526–8. doi: 10.1093/bioinformatics/bty633 30016406

[57] Aktas C. haplotypes: Manipulating DNA sequences and estimating unambiguous haplotype network with statistical parsimony. 2023. Available: https://CRAN.R-project.org/package=haplotypes

[58] Yu G, Smith DK, Zhu H, Guan Y, Lam TT. ggtree: an r package for visualization and annotation of phylogenetic trees with their covariates and other associated data. Methods Ecol Evol. 2016;8(1):28–36. doi: 10.1111/2041-210x.12628

[59] Yu G. aplot: Decorate a “ggplot” with Associated Information. 2020. p. 0.2.3. doi: 10.32614/CRAN.package.aplot

[60] Frichot E, François O. LEA: An R package for landscape and ecological association studies. Methods Ecol Evol. 2015;6(8):925–9. doi: 10.1111/2041-210x.12382

[61] Gain C, François O. LEA 3: Factor models in population genetics and ecological genomics with R. Mol Ecol Resour. 2021;21(8):2738–48. doi: 10.1111/1755-0998.13366 33638893

[62] Patterson N, Price AL, Reich D. Population structure and eigenanalysis. PLoS Genet. 2006;2(12):e190. doi: 10.1371/journal.pgen.0020190 17194218 PMC1713260

[63] Pritchard JK, Stephens M, Donnelly P. Inference of population structure using multilocus genotype data. Genetics. 2000;155(2):945–59. doi: 10.1093/genetics/155.2.945 10835412 PMC1461096

[64] Tracy CA, Widom H. Level spacing distributions and the Bessel kernel. CommunMath Phys. 1994;161(2):289–309. doi: 10.1007/bf02099779

[65] Cattell RB. The Scree Test For The Number Of Factors. Multivar Behav Res. 1966;1(2):245–76. doi: 10.1207/s15327906mbr0102_10 26828106

[66] Paradis E. pegas: an R package for population genetics with an integrated-modular approach. Bioinformatics. 2010;26(3):419–20. doi: 10.1093/bioinformatics/btp696 20080509

[67] Goudet J, Jombart T. hierfstat: Estimation and tests of hierarchical f-statistics. 2022. Available: https://CRAN.R-project.org/package=hierfstat

[68] Kamvar ZN, Tabima JF, Grünwald NJ. *Poppr*: an R package for genetic analysis of populations with clonal, partially clonal, and/or sexual reproduction. PeerJ. 2014;2:e281. doi: 10.7717/peerj.281 24688859 PMC3961149

[69] Kamvar ZN, Brooks JC, Grünwald NJ. Novel R tools for analysis of genome-wide population genetic data with emphasis on clonality. Front Genet. 2015;6:208. doi: 10.3389/fgene.2015.00208 26113860 PMC4462096

[70] Jombart T. adegenet: a R package for the multivariate analysis of genetic markers. Bioinformatics. 2008;24(11):1403–5. doi: 10.1093/bioinformatics/btn129 18397895

[71] Jombart T, Ahmed I. adegenet 1.3-1: new tools for the analysis of genome-wide SNP data. Bioinformatics. 2011;27(21):3070–1. doi: 10.1093/bioinformatics/btr521 21926124 PMC3198581

[72] Weir BS, Cockerham CC. Estimating F-Statistics for the Analysis of Population Structure. Evolution. 1984;38(6):1358. doi: 10.2307/2408641 28563791

[73] Leslie MS, Archer FI, Morin PA. Mitogenomic differentiation in spinner (*Stenella longirostris*) and pantropical spotted dolphins (S. attenuata) from the eastern tropical Pacific Ocean. Marine Mammal Science. 2018;35(2):522–51. doi: 10.1111/mms.12545

[74] Hancock-Hanser BL, Frey A, Leslie MS, Dutton PH, Archer FI, Morin PA. Targeted multiplex next-generation sequencing: advances in techniques of mitochondrial and nuclear DNA sequencing for population genomics. Mol Ecol Resour. 2013;13(2):254–68. doi: 10.1111/1755-0998.12059 23351075

[75] Nykänen M, Kaschner K, Dabin W, Brownlow A, Davison NJ, Deaville R, et al. Postglacial Colonization of Northern Coastal Habitat by Bottlenose Dolphins: A Marine Leading-Edge Expansion? J Hered. 2019;110(6):662–74. doi: 10.1093/jhered/esz039 31211393

[76] Davison AC, Hinkley DV. Bootstrap methods and their applications. Cambridge: Cambridge University Press; 1997. Available: doi: 10.1017/CBO9780511802843

[77] Canty A, Ripley BD. boot: Bootstrap R (s-plus) functions. 2024.

[78] Thulin M. boot.pval: Bootstrap p-Values. 2023. Available: https://CRAN.R-project.org/package=boot.pval

[79] Samsuwan J, Somboonchokepisal T, Akaraputtiporn T, Srimuang T, Phuengsukdaeng P, Suwannarat A, et al. A method for extracting DNA from hard tissues for use in forensic identification. Biomed Rep. 2018;9(5):433–8. doi: 10.3892/br.2018.1148 30402227 PMC6200996

[80] Takahashi M, Kato Y, Mukoyama H, Kanaya H, Kamiyama S. Evaluation of five polymorphic microsatellite markers for typing DNA from decomposed human tissues--correlation between the size of the alleles and that of the template DNA. Forensic Sci Int. 1997;90(1–2):1–9. doi: 10.1016/s0379-0738(97)00129-1 9438360

[81] Ansmann IC, Parra GJ, Lanyon JM, Seddon JM. Fine-scale genetic population structure in a mobile marine mammal: inshore bottlenose dolphins in Moreton Bay, Australia. Mol Ecol. 2012;21(18):4472–85. doi: 10.1111/j.1365-294X.2012.05722.x 22882348

[82] Cipriano F, Hevia M, Iñíguez M. Genetic divergence over small geographic scales and conservation implications for Commerson’s dolphins (*Cephalorhynchus commersonii*) in southern Argentina. Marine Mammal Science. 2010;27(4):701–18. doi: 10.1111/j.1748-7692.2010.00434.x

[83] Chivers SJ, Dizon AE, Gearin PJ, Robertson KM. Small-scale population structure of eastern North Pacific harbour porpoises (*Phocoena phocoena*) indicated by molecular genetic analyses. J Cetacean Res Manage. 2023;4(2):111–22. doi: 10.47536/jcrm.v4i2.847

