# Supplementary figures and images for: Assessment of the genetic diversity of Atlantic bottlenose dolphin (Tursiops truncatus) strandings in the Mississippi Sound (USA)
Source: PLoS One. 2025 Jun 25;20(6):e0314249. doi: 10.1371/journal.pone.0314249 (PMC12194042; doi:10.1371/journal.pone.0314249)

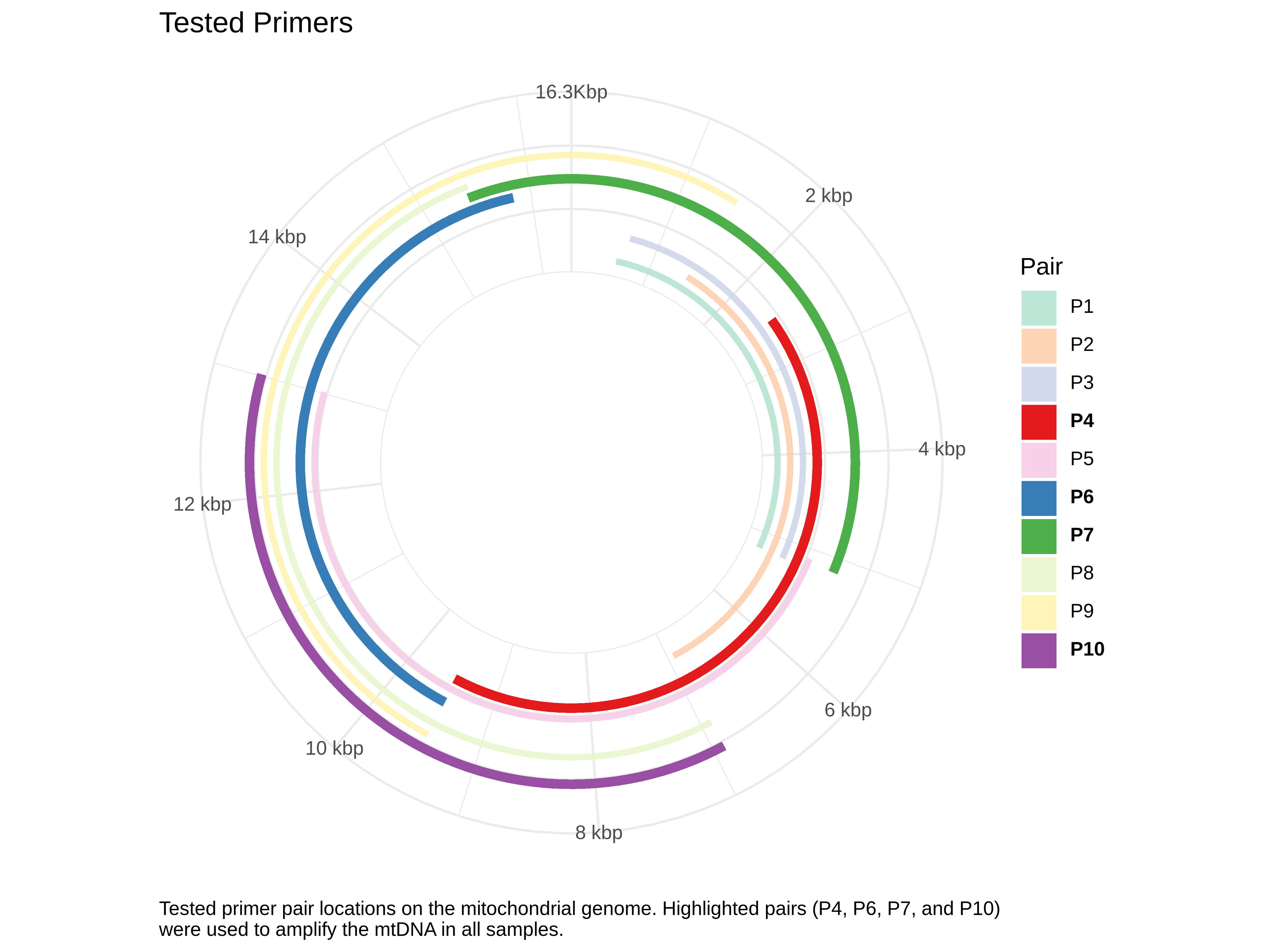

Supplement: S1 Fig — Highlighted pairs (P4, P6, P7, and P10) were used to amplify the mtDNA in all samples. (TIF) [file pone.0314249.s009.tif]

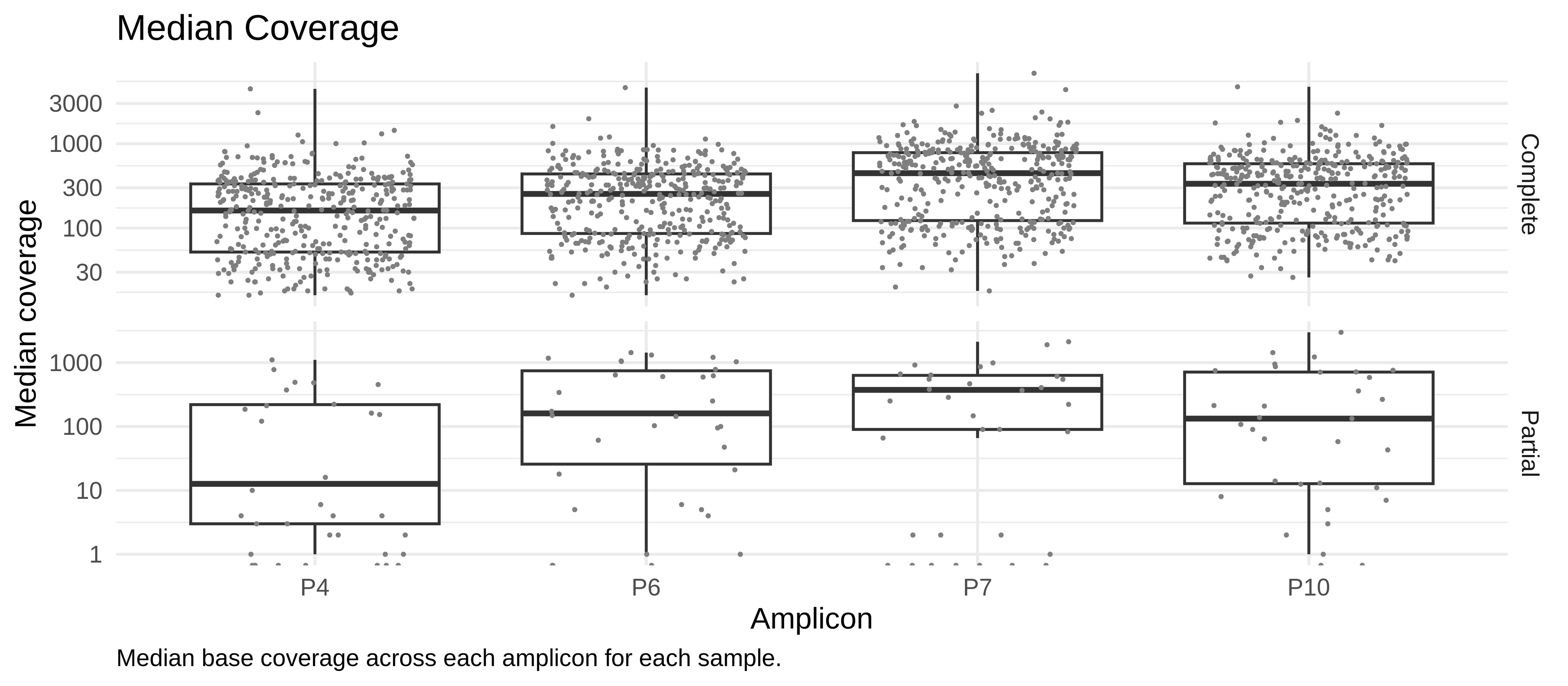

Supplement: S2 Fig — (TIF) [file pone.0314249.s010.tif]

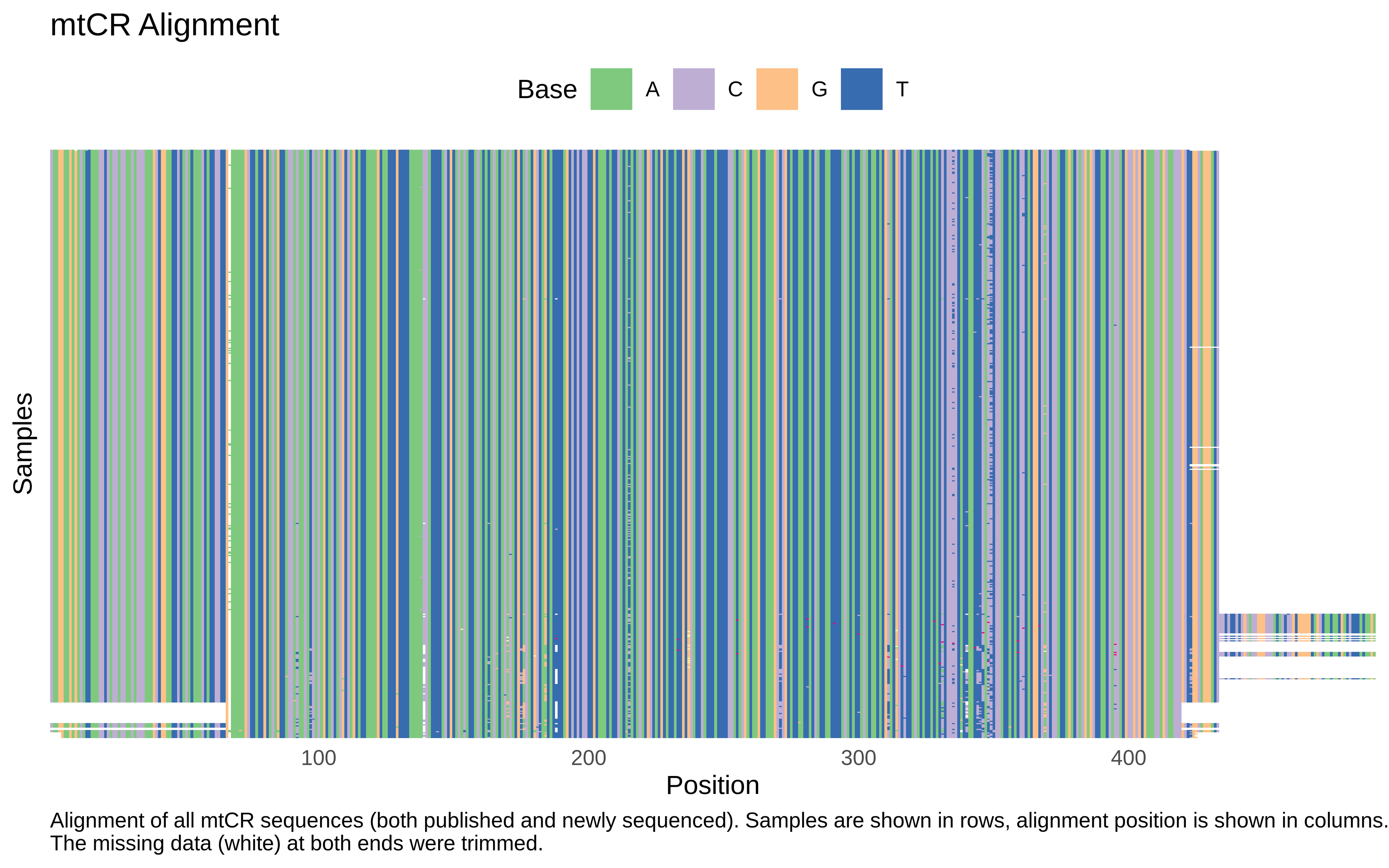

Supplement: S3 Fig — Samples are shown in rows, alignment position is shown in columns. The missing data (white) at both ends were trimmed. (TIF) [file pone.0314249.s011.tif]

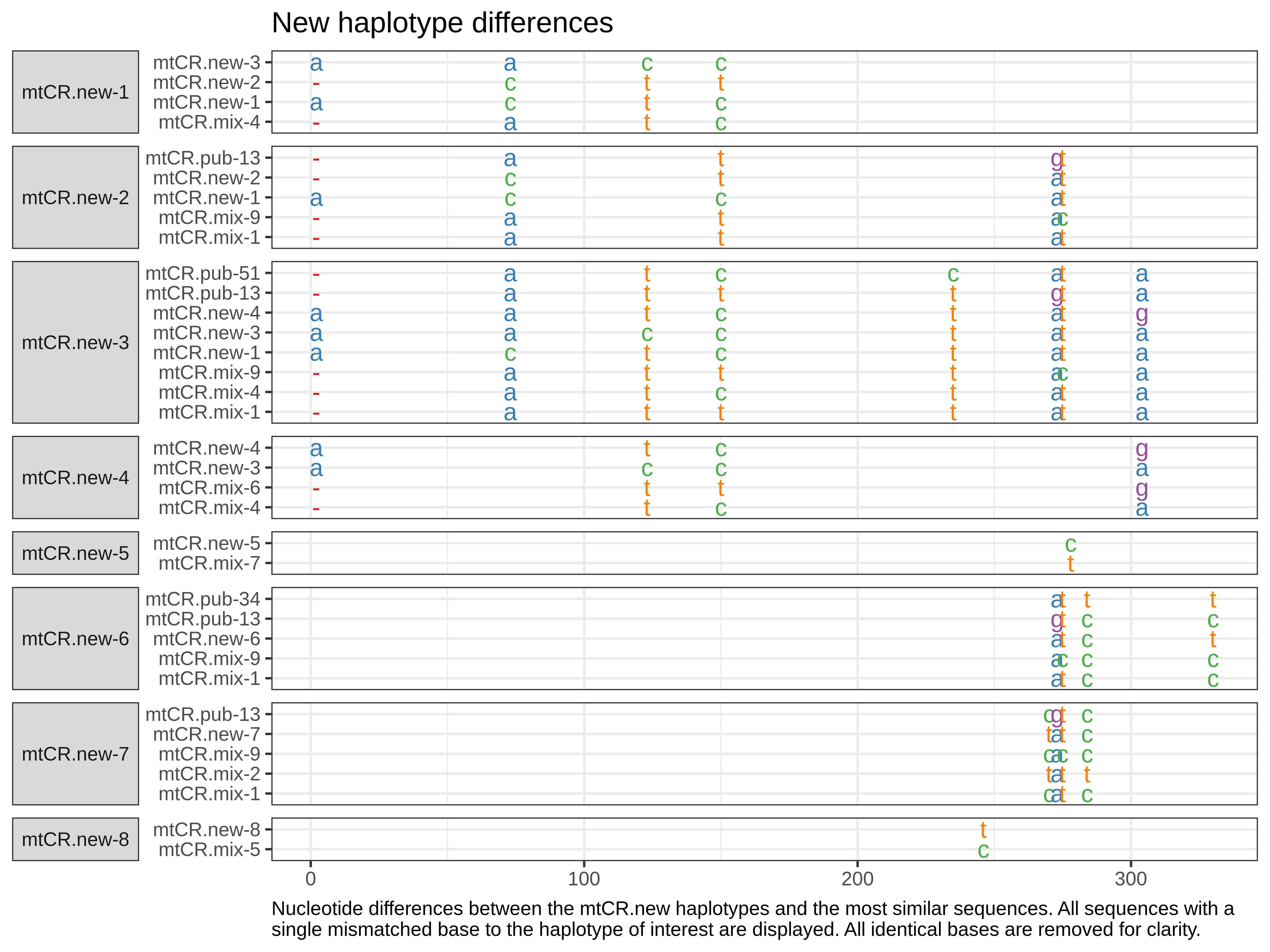

Supplement: S4 Fig — All sequences with a single mismatched base to the haplotype of interest are displayed. All identical bases are removed for clarity. (TIF) [file pone.0314249.s012.tif]

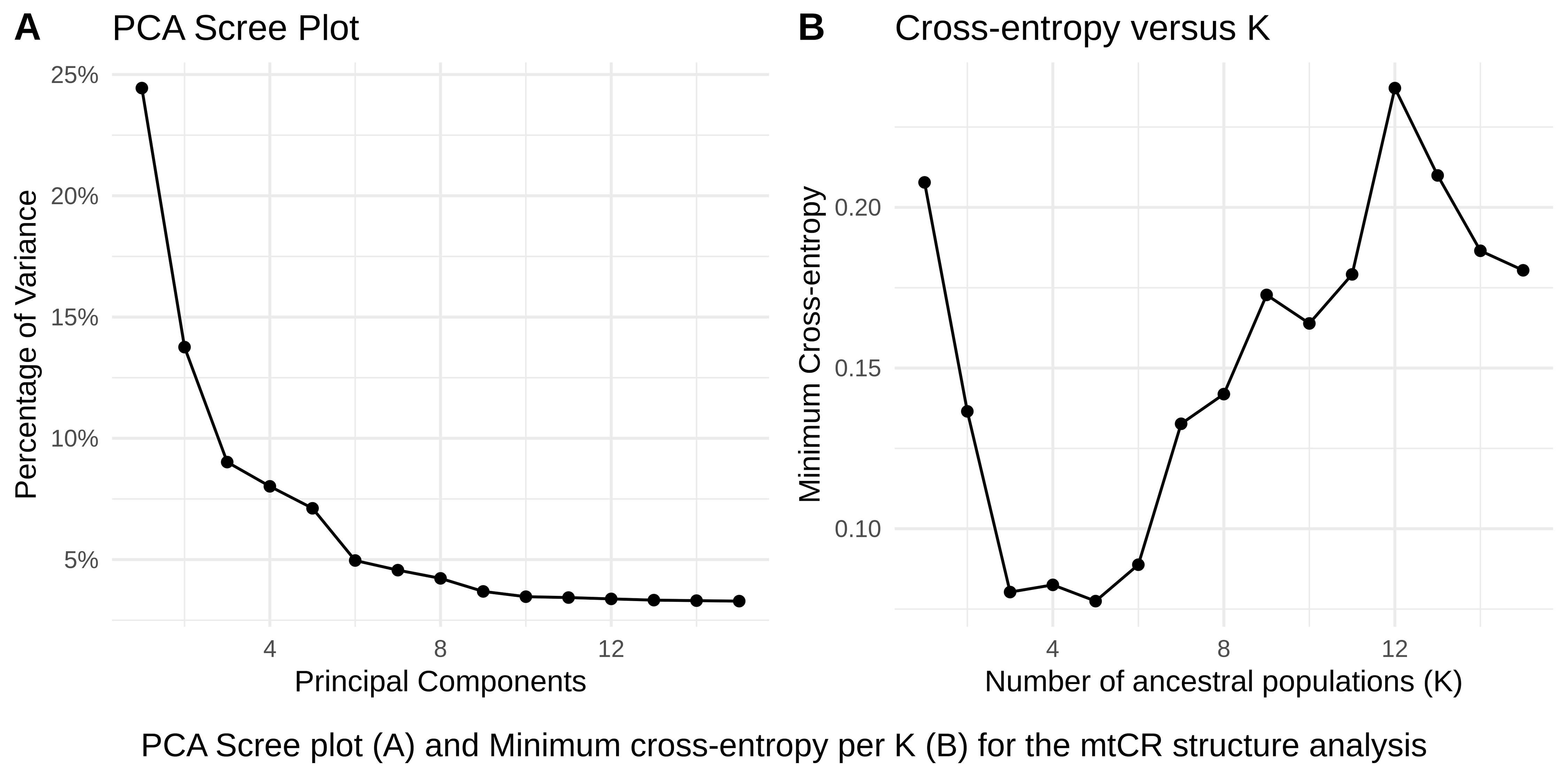

Supplement: S5 Fig — (TIF) [file pone.0314249.s013.tif]

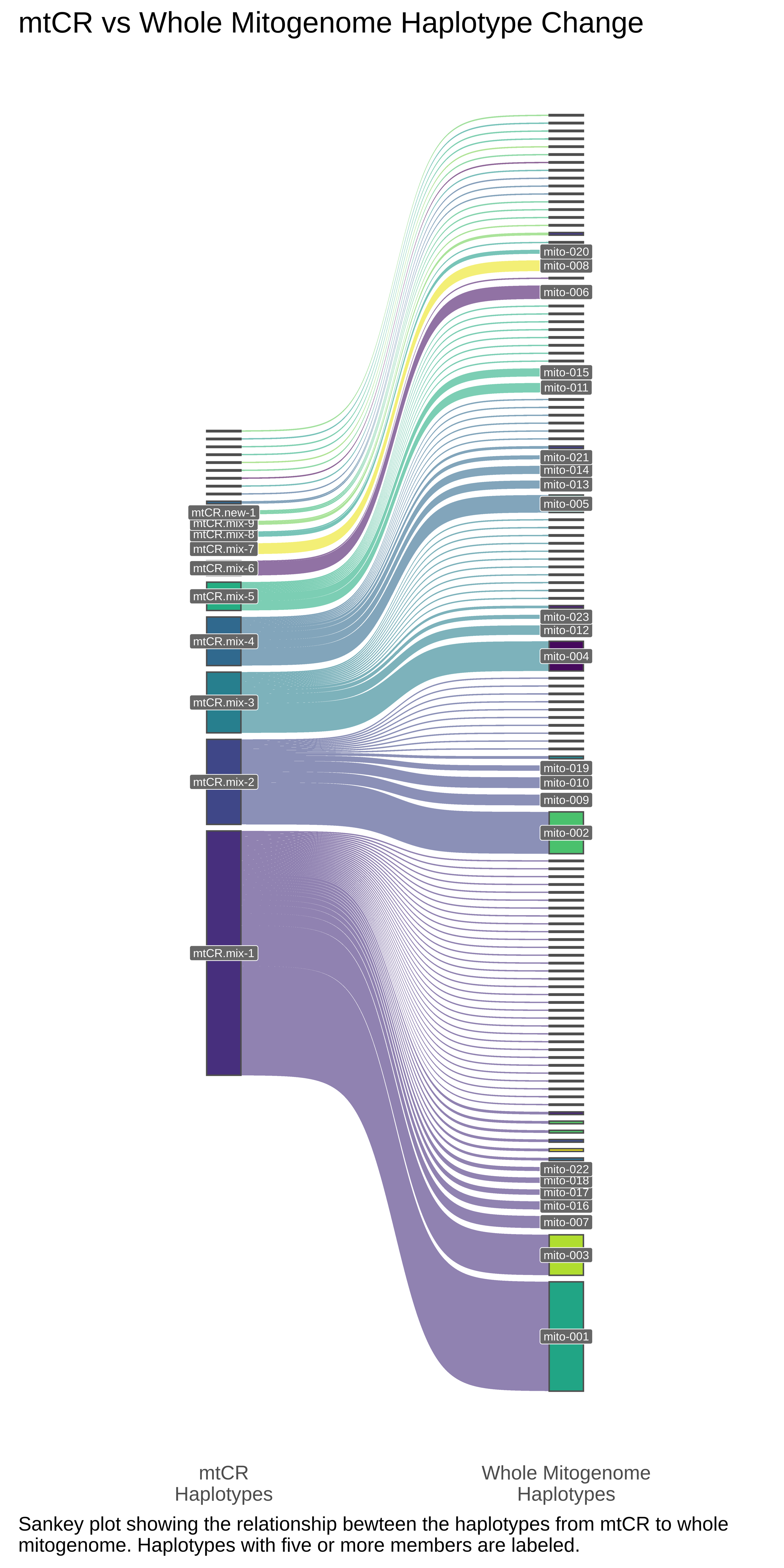

Supplement: S6 Fig — Haplotypes with five or more members are labeled. (TIF) [file pone.0314249.s014.tif]

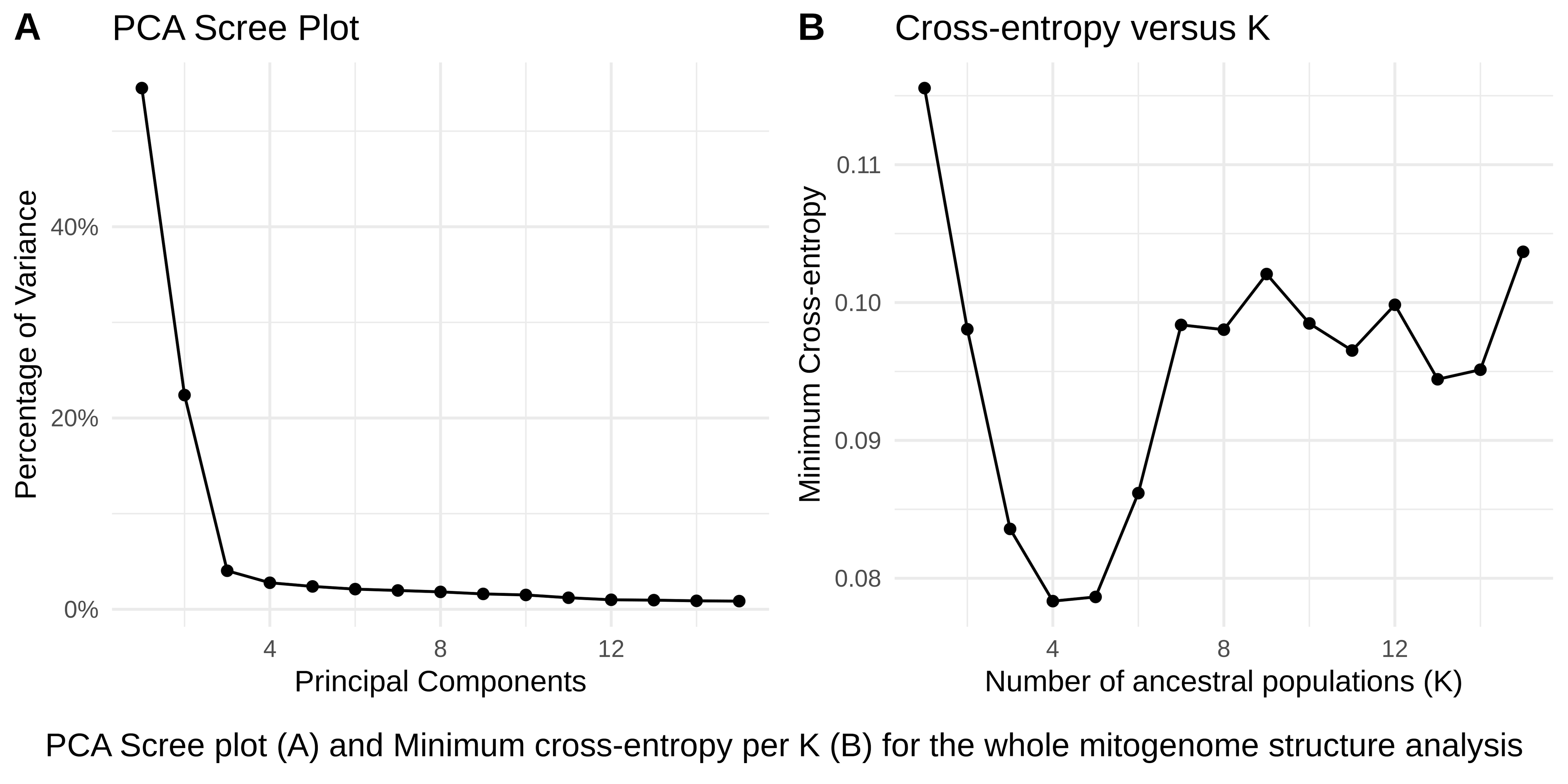

Supplement: S7 Fig — (TIF) [file pone.0314249.s015.tif]

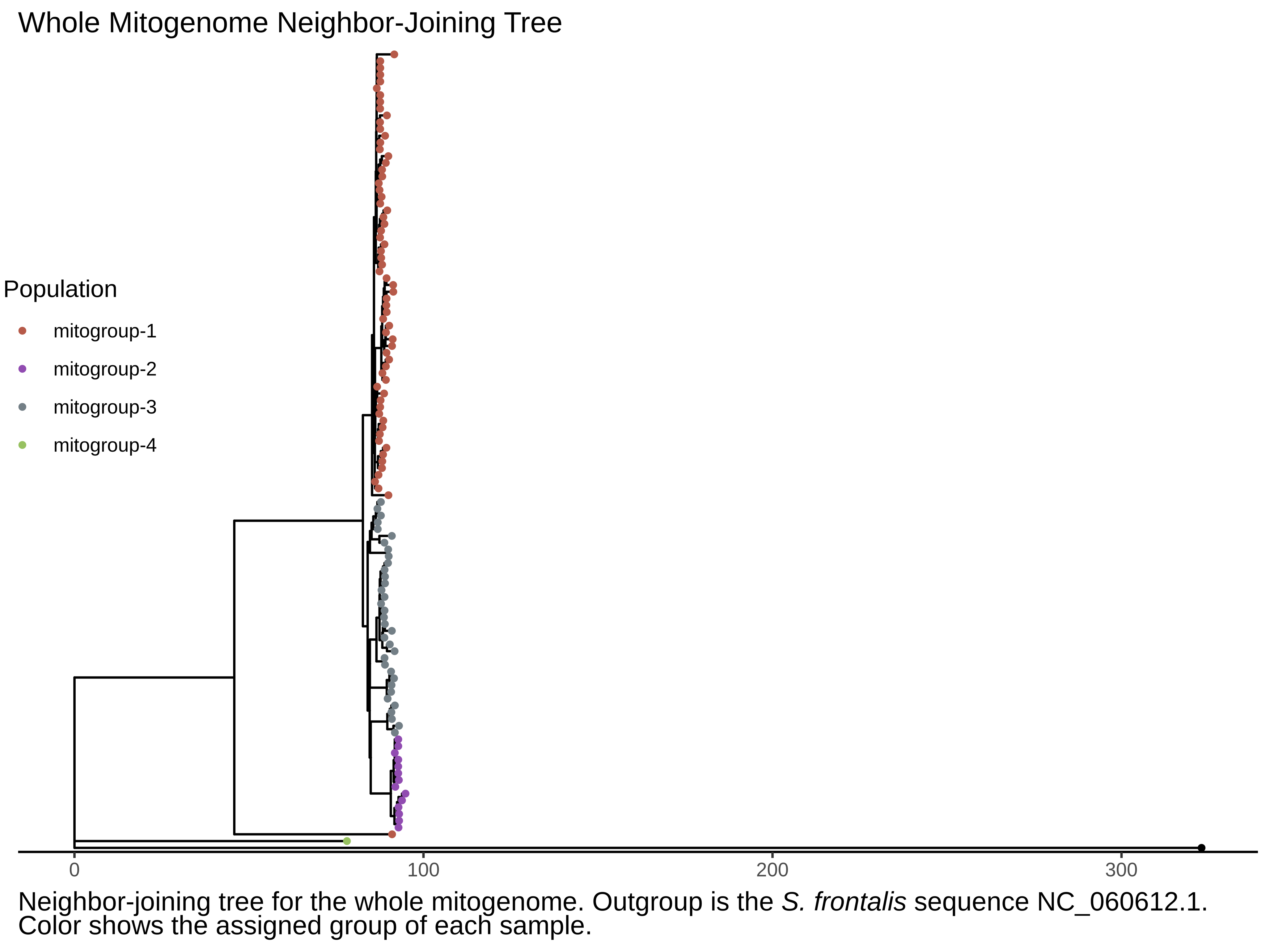

Supplement: S8 Fig — Outgroup is the S. frontalis sequence NC_060612.1. Color shoe the assigned group of each sample. (TIF) [file pone.0314249.s016.tif]
